# Engineering Solutions for Cranio-Maxillo-Facial Rehabilitation and Oro-Dental Healthcare

**DOI:** 10.1155/2019/5387305

**Published:** 2019-06-18

**Authors:** Ziyad S. Haidar, Lucy Di‐Silvio, Ziad E. F. Noujeim, John E. Davies, Frédéric Cuisinier, Avijit Banerjee

**Affiliations:** ^1^BioMAT'X, Universidad de los Andes, Santiago, Chile; ^2^Programa de Doctorado en BioMedicina, Facultad de Medicina, Universidad de los Andes, Santiago, Chile; ^3^Centro de Investigación e Innovación Biomédica, Universidad de los Andes, Santiago, Chile; ^4^Facultad de Odontología, Universidad de los Andes, Santiago, Chile; ^5^Department of Biomaterials and Biomimetics, King's College London, London, UK; ^6^Department of Oral Medicine and Maxillofacial Radiology, Faculty of Dental Medicine, Lebanese University, Beirut, Lebanon; ^7^Institute of Biomaterials and Biomedical Engineering, University of Toronto, Toronto, Canada; ^8^Laboratoire Bioingénierie et Nanosciences, Université de Montpellier, Montpellier, France; ^9^Centre for Oral and Craniofacial Sciences, King's College London, London, UK

Oro-dental health is an essential component of good overall health and well-being, and consequently, good oral and dental health is indeed a fundamental human right, part of the main role, and goal of the dental profession with all its interdisciplinary and multidisciplinary subspecialties to help the population achieve a stable and healthy craniomaxillofacial complex. Hence, identifying challenges and opportunities needs to be in the forefront of our priorities, especially considering the mounting ageing population and swelling demand for oro-dental healthcare, products, services, and alternative solutions. It is noteworthy herein that this agrees with and supports the global 2020 vision of the FDI (Fédération Dentaire Internationale) World Dental Federation, the world's leading dental professional organization, on oral and dental health, where it is proposed and urged to expand the role of existing oral and dental healthcare professionals and foster fundamental research and translational technologies, to better mitigate the impacts of socioeconomic dynamics, a cornerstone for the projection of this special issue.

In brief, innovative engineering solutions that incorporate advanced biomaterials, nanobiotechnology, three-dimensional printing, computer assistance, and robotic systems offer huge potential for augmenting and improving the functional and esthetic craniomaxillofacial and oro-dental health profile of patients. A good example, perhaps, is nanoDentistry, clearly multidisciplinary and interdisciplinary, building on existing knowledge and accruing expertise in different scientific and technological fields, seeking persistent refinement of traditional approaches, via the development and/or incorporation of advanced biomaterials, new *functional* tools, and pharmacological formulations to improve overall oro-dental practice and care. While slowly evolving, nanoDentistry is expected to provide dentists and surgeons with more precision-made and tailored materials, drugs, and equipment, by which safety, esthetics, function/efficacy, and patient compliance are enhanced. Due to the complex nature of such “outside-the-box” (or even “no-box”) healthcare-related engineering *problem–solution* technologies, they have attracted experts from physics, chemistry, biology, materials science, pharmaceutics, robotics, and bioengineering, as well as the industry. The ultimatum is improved overall healthcare and well-being with a positive impact on socioeconomics and quality of life.

Hence, this special issue is intended to bring together a collection of original and state-of-the-art works in oro-dental and craniomaxillofacial healthcare and related topics to exhibit the latest ideas, concepts, findings, achievements, and future projections and promote awareness of the rapidly evolving and enabling multidisciplinary technology, thereby encouraging a fruitful dialogue to bridge the gap between engineering and dentistry (including subspecialties, extending to the head and neck) for research and innovation collaboration across the fields to address the critical and urgent biodental/biomedical concerns. In particular, this special issue assembles six original contributions presenting recent advances in biomechanical properties of periodontal tissues and metal-based biomaterials, as well as stimulating mathematical modeling methods as fundamental and expedited analytical tools, for clinical use. Different areas of engineering solutions, once more, clearly support the previously mentioned growing need and our hope to bridge the disciplinary communication and collaboration gap, through this contribution.

In no particular order, the paper by C. Sinescu et al. entitled “Mechanical Properties of the Periodontal System and of Dental Constructs Deduced from the Free Response of the Tooth” explores the biomechanical behavior of the periodontal ligament (PDL), the fibrous connective and neurovascular tissue that connects the dentition (cementum part of the root of an individual tooth) to the surrounding alveolar bone, for proper and healthy support of mastication and nutritional intake, among other key oro-dental functions detrimental for an overall general health, well-being, and quality of life. By analyzing clinical and mathematical studies, the authors introduce a new method for the evaluation of biomechanical and *elastometric* parameters of the tooth-PDL system, using an ultrasound probe, in different clinical scenarios and conditions. This study presents a linear model in good accordance with physical phenomena that would help the dentist and/or prosthodontist (oro-dental rehabilitation specialist) provide better prosthesis (such as crowns and bridges) and postoperative care for the patient, to help prevent periodontal and/or masticatory diseases or at least arrest the adverse consequences on the well-being of the patient.

L. Bai et al. in the paper entitled “Mechanical Characterization of 3D-Printed Individualized Ti-Mesh (Membrane) for Alveolar Bone Defects” describe the importance of sufficient bone volume in implant dentistry, in both the horizontal and vertical dimensions, and how it plays a vital role in achieving long-term esthetic and functional results. Herein, using computer-assisted design and additive manufacturing technology, a custom-made (personalized/case-by-case) titanium (Ti) mesh is developed. It is deemed superior to conventional and commercially available meshes, taking into consideration, via three-dimensional finite element analysis, the effect of pore size and thickness on the mechanical properties of the Ti mesh, as well as variances in size and anatomical location of bone defects. Clinically, this original effort might eventually translate to shortening the duration of the surgical procedure(s) as well as minimizing if not alleviating or eliminating the risk of postoperative infections, a *fine* example of multidisciplinary healthcare engineering research.

The paper entitled “Bone Loss around Dental Implants 5 Years after Implantation of Biphasic Calcium Phosphate (HAp/*β*TCP) Granules” by V. Klimecs et al. deals with implant dentistry as well and addresses the issue of alveolar bone loss around dental implants, clinically, and its impact on dental implant therapy failure. For this purpose, the authors developed and used *combinatorial* bioceramic granules as a filler biomaterial in eighteen patients suffering peri-implantitis, an infectious disease that causes an inflammatory process in soft tissues and bone loss around an osseointegrated implant. Clinical evaluation and radiological (3D cone-beam computed tomography) measurements were done, after a 5-year period, to compare the situation before and after treatment of peri-implantitis with the use of different bioceramic materials (pure calcium hydroxyapatite *vs.* combined hydroxyapatite and *β*-tricalcium phosphate). This is a registered clinical trial, providing densitometry and mineralization data of bone structures.

On the contrary, S. Wang et al., in the article entitled “Automatic Analysis of Lateral Cephalograms Based on Multiresolution Decision Tree Regression Voting,” developed a fully automatic system to aid in the analysis of cephalograms, a traditional, conventional, and standard two-dimensional radiological X-ray of the craniofacial area (lateral side of the head) often used in orthodontic and orthognathic areas to assess and predict craniofacial growth. It is also commonly used for clinical orthodontic diagnosis and oromaxillofacial treatment planning purposes. Lateral cephalograms often require a specialist to analyze and identify specific landmarks. Herein, a new framework for landmark detection in lateral cephalograms with low-to-high resolutions is designed. The algorithmic method employs multiscale decision tree regression voting in landmark detection, in each image resolution (low vs. high). Patch feature extraction is based on scale-invariant feature transform algorithms. Basically, information can be extracted to predict the positions of the anatomical structure involving the specific landmarks (45, double the landmarks often detected by benchmark databases), with a decent average of 72% successful detection rate within a precision range of 2.0 mm. This is another *fine* example of “outside-the-box” multidisciplinary and multinational collaborative novelty for engineering solutions in health.

Likewise, the paper by D. Xu et al. entitled “Interactive Compliance Control of a Wrist Rehabilitation Device (WR*e*D) with Enhanced Training Safety” is an inordinate illustration of different disciplines and areas of research coming together. It deals with human-robot interactive tasks (and interaction control), robot-assisted rehabilitation solutions, and physical therapy, to improve the safety and efficacy of training and compliance adaptation of interaction, as are *vital* for enabling robotic or robot-specific movements to better suit the varying and/or personalized requirements of individual patients. Herein, this work proposes an interactive compliance control scheme on a wrist rehabilitation device, with satisfactory trajectory tracking responses. Interestingly, this interactive compliance control method can adaptively adjust the range of training motions and encourage active engagement from human users simultaneously, opening doors wide for more clinical applications. It is perhaps worth mentioning, for the interested reader, that the interactive compliance control of the wrist rehabilitation device was achieved based on an admittance law (where the device deviates from the reference trajectory in the presence of patient-robot interaction nevertheless is also still following the reference trajectory—*read article for more inspiring details!*) due to the availability of direct measurement of the interaction torque.

Last but not least, G. P. Panotopoulos et al. revisit the controversial issue of cell migration and cell-to-cell communications in the article entitled “Non-Motile Single-Cell Migration as a Random Walk in Non-Uniformity: The “Extreme Dumping Limit” for Cell-to-Cell Communications.” Single-cell movement as a random walk in an external potential, observed within the extreme dumping limit (defined herein as the extreme nonuniform behavior observed for cell responses and cell-to-cell communications), is mathematically modeled in this work. The authors attempt to solve the Fokker–Planck equation in order to compute higher “moments” of the displacement of the cell and then build experimentally measurable quantities. Seemingly, the dynamics depend, every time, on the external force applied, leading to predictions distinct from standard results of a free Brownian particle. This demonstrates that cell migration viewed as a stochastic process is still compatible with biological and experimental observations *without* the need to rely on more complicated or sophisticated models previously proposed in the literature. The derived model and equation could be potentially beneficial to explain cellular migration during relevant biological processes, including wound healing, inflammation, and embryonic development, via a minimalistic approach.

Finally, while we believe and aspire that this special issue will be useful and inspiring for scientists, researchers, engineers, and clinical practitioners involved in healthcare engineering solutions, it is recommended to pay special attention to the potentially impactful role of applied bio-dental tissue engineering, drug/gene delivery (controlled and metered systems) and cell therapy, image-guided and -assisted surgery, and nanoDentistry and green dentistry, towards developing better solutions for problems and conditions of the oro-dental and cranio-maxillo-facial complex.

